# Development and Validation of a Novel Model for Predicting Prognosis of Non-PCR Patients After Neoadjuvant Therapy for Breast Cancer

**DOI:** 10.3389/fonc.2021.675533

**Published:** 2021-09-01

**Authors:** Yongqiang Yu, Si Wu, Hui Xing, Mengxue Han, Jinze Li, Yueping Liu

**Affiliations:** Department of Pathology, The Fourth Hospital of Hebei Medical University, Shijiazhuang, China

**Keywords:** neoadjuvant therapy, R software, pathologic complete response, tumor-infiltrating lymphocytes, residual tumor burden

## Abstract

**Purpose:**

Pathologic complete response (pCR) after neoadjuvant therapy is an important indicator of long-term prognosis and the primary endpoint of many neoadjuvant studies. For breast cancer patients who do not achieve pCR, prognostic indicators related to prognosis are particularly important. This study is constructing a prediction model with more accurate and reliable prediction results by combining multiple clinicopathological factors, so as to provide a more accurate decision-making basis for subsequent clinical treatment.

**Patients and Methods:**

In this study, 1,009 cases of invasive breast cancer and surgically resected after neoadjuvant therapy from 2010 to 2017. All indicators in this trial were interpreted in a double-blind manner by two pathologists with at least 10 years of experience, including histological grading, Tils, ER, PR, HER2, and Ki67. The prediction model used R language to calculate the calibration degree and ROC curve of the prediction model in the training set and validation set.

**Results:**

Through univariate survival analysis, the results showed histological grade (P=0.037), clinical stage (P<0.001), HER2 (P=0.044), RCB class (P<0.001), Tils (P<0.001), lymph node status (P =0.049), MP grade (P=0.013) are related to OS in non-PCR patients after neoadjuvant. Data were analyzed by substituting in a multivariate analysis, and the results were that clinical stage, HER2, RCB grading, and Tils grading were correlated with OS in non-PCR patients after neoadjuvant therapy for breast cancer. Among all cases in the training set, the prediction model predicted that the 3-year survival AUC value was 0.95 and 5-year survival AUC value was 0.79, and the RCB classification of 3-year survival and 5-year survival were 0.70 and 0.67, respectively, which proved that the prediction model could predict the OS of non-PCR patients after neoadjuvant therapy for breast cancer more accurately than the RCB classification, and showed the same results in HR, HER2+, and TN classifications. It also showed the same results in validation set.

**Conclusion:**

These data indicate that the predicted values of the prediction model developed in this study match the actual survival rates without underestimating the mortality risk and have a relatively accurate prediction effect.

## Introduction

Neoadjuvant therapy (NAC) allows inoperable patients with advanced breast cancer to achieve downstaging, which in turn makes the patient operable and facilitates breast-conserving surgery (1). As breast cancer treatments continue to be explored, neoadjuvant therapy is also widely used in early-stage breast cancer, which makes treatment outcome of neoadjuvant therapy an important indicator of patient prognosis (2).

Pathologic complete response (pCR) after neoadjuvant therapy is an important indicator of long-term prognosis and the primary endpoint of many neoadjuvant studies, and patients who achieve pCR have a good long-term prognosis (3, 4). For breast cancer patients who do not achieve pCR, prognostic indicators related to prognosis are particularly important. With increasing research on the immune microenvironment and evidence of clinical relevance of tumor-infiltrating lymphocytes (Tils) in breast cancer, Tils have shown potential predictive value for prognosis, especially in triple-negative and HER2-overexpressing breast cancer, with results showing a positive correlation between the degree of Tils infiltration and prognosis (5, 6). The residual cancer burden (RCB) scoring system can be used as an independent prognostic factor for patient survival, with worse prognosis as the RCB grade increases (7). However, the RCB scoring system only combines some pathological factors after neoadjuvant therapy. In the ongoing study on prognostic factors correlated with breast cancer after neoadjuvant therapy, it has been shown that individual pathological factors have more or less limitations, and the combination of multiple prognostic indicators can provide a better prognosis and guide the decision-making for subsequent clinical treatment (8). The predictive model in this study is an algorithmic model that integrates prognostic-related factors and is widely used in the prognostic analysis of various cancer types to provide clinical decision-making (9–13). In order to better predict the prognosis of non-pCR patients after neoadjuvant therapy for breast cancer, this study counted the prognostic-related factors after neoadjuvant therapy for breast cancer and constructed a prediction model using R software, with the aim of constructing a prediction model with more accurate and reliable prediction results by combining multiple clinicopathological factors, so as to provide a more accurate decision-making basis for subsequent clinical treatment.

## Materials and Methods

### General Information

In this study, 1,009 cases of invasive breast cancer diagnosed by core needle biopsy and surgically resected after neoadjuvant therapy from 2010 to 2017 were selected from the Fourth Hospital of Hebei Medical University, and all patients were female. Clinicopathological data of patients were collected: age, gender, clinical stage, histological grading, lymphatic vascular tumor thrombosis, lymph node involvement, tumor-infiltrating lymphocytes (Tils), estrogen receptor (ER), progesterone receptor (PR), and HER2 (**Table 1**). Inclusion criteria are as follows: ① invasive breast cancer diagnosed by core needle biopsy before neoadjuvant therapy; ② complete immunohistochemical indexes; ③ standardized neoadjuvant therapy (four to six cycles); ④ complete follow-up information. Exclusion criteria are the following: ① non-invasive breast cancer diagnosed by core needle biopsy before neoadjuvant therapy; ② incomplete immunohistochemistry results; ③ no neoadjuvant therapy for physical tolerance or other reasons; ④ no definite follow-up information. All indicators in this trial were interpreted in a double-blind manner by two pathologists with at least 10 years of experience, including histological grading, Tils, ER, PR, HER2, and Ki67. According to the expression status of hormone receptors, we have further classified breast cancer: hormone receptor positive (HR): ER positive, HER2 negative; human epidermal growth factor 2 positive (HER2+): HER2 positive; triple-negative breast cancer (TN): ER negative, PR negative, HER2 negative.

**Table 1:** Baseline characteristics of patients.

	Total	Training	Validation	P
Age (years)		N = 505 (100(%))	504	
<50	512 (51%)	259 (51.29%)	253 (50.2%)	0.78
≥50	497 (49%)	246 (48.71%)	251 (49.8%)	
Histological grade		N = 505 (100(%))		
I	52 (5%)	25 (4.95%)	27 (5.36%)	0.96
II	686 (68%)	344 (68.12%)	342 (67.86%)	
III	271 (27%)	136 (26.93%)	135 (26.79%)	
Angioma thrombus		N = 505 (100(%))		
−	717 (71%)	357 (70.69%)	360 (71.43%)	0.85
+	292 (29%)	148 (29.31%)	144 (28.57%)	
Clinical stage		N = 505 (100(%))		
I	64 (6%)	39 (7.72%)	25 (4.96%)	0.21
II	265 (26%)	138 (27.33%)	127 (25.2%)	
III	562 (56%)	269 (53.27%)	293 (58.13%)	
IV	118 (12%)	59 (11.68%)	59 (11.71%)	
Lymph node status		N = 505 (100(%))		
−	184 (18%)	93 (18.42%)	91 (18.06%)	0.95
+	825 (82%)	412 (81.58%)	413 (81.94%)	
Mitosis		N = 505 (100(%))		
1	317 (31%)	158 (31.29%)	159 (31.55%)	0.33
2	629 (62%)	321 (63.56%)	308 (61.11%)	
3	63 (6%)	26 (5.15%)	37 (7.34%)	
MP class		N = 505 (100(%))		
1	67 (7%)	24 (4.75%)	43 (8.53%)	0.09
2	231 (23%)	123 (24.36%)	108 (21.43%)	
3	518 (51%)	259 (51.29%)	259 (51.39%)	
4	190 (19%)	96 (19.01%)	94 (18.65%)	
5	3 (0%)	3 (0.59%)	0 (0%)	
RCB class		N = 505 (100(%))		
I	52 (5%)	26 (5.15%)	26 (5.16%)	0.49
II	511 (51%)	265 (52.48%)	246 (48.81%)	
III	446 (44%)	214 (42.38%)	232 (46.03%)	
ER		N = 505 (100(%))		
−	289 (29%)	137 (27.13%)	152 (30.16%)	0.32
+	720 (71%)	368 (72.87%)	352 (69.84%)	
PR		N = 505 (100(%))		
−	363 (36%)	174 (34.46%)	189 (37.5%)	0.35
+	646 (64%)	331 (65.54%)	315 (62.5%)	
KI67		N = 505 (100(%))		
−	140 (14%)	79 (15.64%)	61 (12.1%)	0.12
+	869 (86%)	426 (84.36%)	443 (87.9%)	
HER2		N = 505 (100(%))		
−	663 (66%)	341 (67.52%)	322 (63.89%)	0.25
+	346 (34%)	164 (32.48%)	182 (36.11%)	
Molecular		N = 505 (100(%))		
HR	746 (74%)	69 (13.66%)	82 (16.27%)	0.43
HER2+	151 (15%)	382 (75.64%)	364 (72.22%)	
TN	112 (11%)	54 (10.69%)	58 (11.51%)	
Tils		N = 505 (100(%))		
Low	213 (21%)	99 (19.6%)	114 (22.62%)	0.5
Moderate	604 (60%)	308 (60.99%)	296 (58.73%)	
High	192 (19%)	98 (19.41%)	94 (18.65%)	
N	1,009	505	504	

ER, estrogen receptor; PR, progesterone receptor; HER2, human epidermal growth factor 2; Tils, tumor-infiltrating lymphocytes; −, negative; +, positive; HR, hormone receptor positive; HER2+, human epidermal growth factor 2 positive; TN, triple-negative breast cancer.

### Preparation of Tissue Specimens

All specimens in this study were surgically resected specimens after neoadjuvant therapy. All sections were greater than 100 cancer cells. All breast cancer specimens were fixed with 4% neutral (phosphate buffered) formaldehyde fixative within a specified time period (within 30 min) after isolation. All immunohistochemically stained (IHC) sections in this study had a section thickness of 4 µm, and HE-stained sections were available as a comparison for all assays.

### Interpretation of Hormone Receptor Expression

According to the detection standards of ER and PR in “Estrogen and Progesterone Receptor Testing in Breast Cancer: ASCO/CAP Guideline Update”, the definition of positive for ER and PR detection means that 1 to 100% of the nucleus of the sample is positively stained. If <1 or 0% of tumor cell nuclei are immunoreactive, the sample is considered ER or PR negative (14).

HER2 was interpreted according to “Human Epidermal Growth Factor Receptor 2 Testing in Breast Cancer: American Society of Clinical Oncology/College of American Pathologists Clinical Practice Guideline Focused Update”. HER2 interpretation results are 0, 1+, 2+, 3+; for IHC results for 2+ cases, FISH should be used for further testing, or different tissue blocks can be selected for retesting or sent to a laboratory with better conditions for testing. In the HER2 results, the 0, 1+, and 2+ FISH test results were determined as negative expression, and the IHC results of 3+ and 2+ FISH results were determined as positive expression (15).

According to the “Assessment of Ki67 in Breast Cancer: Recommendations from the International Ki67 in Breast Cancer Working Group”, the Ki67 interpretation method is to select at least three high-power fields (×40 objective lens, at least 1,000 tumor cells) for each slice for counting. Any numerical value of nuclear positive staining is considered positive. In this study, <20% of cases were judged as low expression of KI67, and ≥20% of cases were judged as high expression of KI67 (16).

### Interpretation of Tumor-Infiltrating Lymphocytes

Tils require at least one HE slice (4–5 μm) for each case. The interpretation criterion is the percentage of the area of tumor-infiltrating lymphocytes in the interstitial area of the invasive tumor border to the total interstitial area of the invasive tumor border. The range of interpretation was within the boundary of invasive tumor [take the tumor border as the midline, and within 1 mm (a 20× high-power field of view) as the tumor border], exclude necrosis, degeneration, ductal carcinoma *in situ* of the breast, and tumor-infiltrating lymphocytes around normal lobules. The final evaluation result should be the average value of all regions instead of the hot spot value. The evaluation result is rounded to the nearest 5–10%. This study divides the interpretation results of Tils into three levels: low invasion is <10%, intermediate invasion 10–40%, and high invasion is ≥40% (6).

### Interpretation of Residual Tumor Burden

The interpretation of RCB requires statistics on the long and wide diameters of the largest cross-section of the tumor bed, the percentage of invasive cancer in the tumor bed area, the number of metastatic lymph nodes, and the maximum diameter of lymph node metastasis.

$$\text{RCB}=1.4\;(\text{invasive cancer Percentage}\times \text{tumor bed tumor size}{)}^{0.17}+\;[4\;(1-0.\;75^{\text{number of metastatic lymph nodes}})\times \text{maximum diameter of lymph node metastasis}{]}^{0.17}.$$

### Statistical Methods

In this study, data analysis was performed by SPSS and R software. All data were randomly divided into the training sets (505 cases) and validation sets (504 cases). Chi-square test was used to calculate whether there were statistically significant differences between the two groups. The pathological indicators correlated with prognosis were selected by Kaplan-Meier and COX multivariate survival analysis. Significant indicators from the multivariate COX regression analysis were included in the prediction model development options, programmed using R software, and finally produced into a prediction model to assess the prognostic outcome of non-PCR patients after neoadjuvant therapy for breast cancer. Each factor corresponded to one point, the scores of the four factors were summed to give a total score, and finally the corresponding 3-year and 5-year survival rates were calculated by using the R language formula. In this study, the prediction model used R language to calculate the calibration degree and ROC curve of the prediction model in all cases, as well as HR, HER2, and TN in the training set and validation set, and the accuracy of the prediction model was tested by the prediction model calibration degree and prediction model ROC curve.

## Results

A total of 1,009 cases suffering from breast cancer and undergoing surgical resection after neoadjuvant therapy from 2010 to 2017 at the Fourth Hospital of Hebei Medical University were selected for this study, with a maximum follow-up time of 115 months, and all patients were female, with a mean patient age of 50 years (24–72 years) and a median survival time of 47 months (12–115 months). All data were randomly divided into the training set (505) and validation set (504), and no statistically significant difference between the two groups was demonstrated by chi-square test (all P values > 0.05).

**Figure 1:**
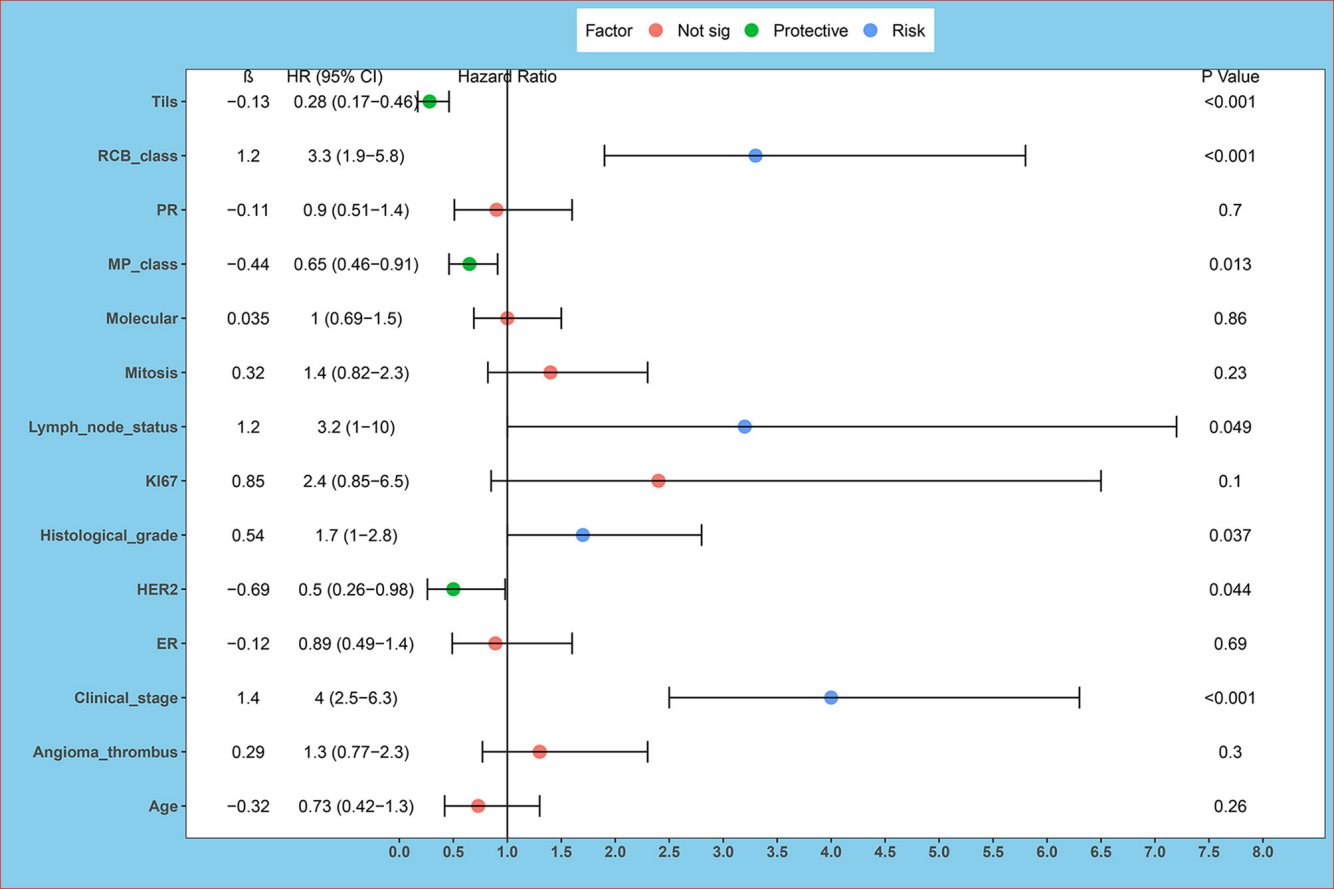
Univariate survival analysis forest plot. ER, estrogen receptor; PR, progesterone receptor; HER2, human epidermal growth factor 2; Tils, tumor-infiltrating lymphocytes. Univariate survival analysis showed histological grade (P = 0.037), clinical stage (P < 0.001), HER2 (P = 0.044), RCB class (P < 0.001), Tils (P < 0.001), lymph node metastasis (P = 0.049), MP grade (P = 0.013) are related to OS in non-PCR patients after neoadjuvant.

Through univariate survival analysis, the results showed histological grade (P=0.037), clinical stage (P<0.001), HER2 (P=0.044), RCB class (P<0.001), Tils (P<0.001), lymph node metastasis (P =0.049), MP grade (P=0.013) were related to OS in non-PCR patients after neoadjuvant. Univariate survival analysis showed that histological grading, clinical stage, HER2, RCB, Tils, lymph node metastasis, and MP grading were correlated with OS in non-PCR patients after neoadjuvant therapy. High histologic grade, high clinical stage, negative HER2 expression, high RCB grade and low Tils, lymph node metastasis, and low MP grade were correlated with poor prognosis (**Table 2** and **Figure 1**).

**Table 2:** Univariate and multivariate survival analysis for OS.

	Univariate		Multivariate	
	HR (95% CI for HR)	p.value	HR (95% CI for HR)	p.value
Histological Grade	1.7 (1.0–2.8)	0.037	*	*
Clinical stage	4 (2.5–6.3)	2.10E-09	3.6550 (2.3062–5.7926)	3.46e-08
Age	0.73 (0.42–1.3)	0.26	*	*
Angioma thrombus	1.3 (0.77–2.3)	0.3	*	*
Lymph node status	3.2 (1–10)	0.049	*	*
RCB class	3.3 (1.9–5.8)	2.50E-05	2.1608 (1.1393–4.0981)	0.01831
ER	0.89 (0.49–1.6)	0.69	*	*
PR	0.9 (0.51–1.6)	0.7	*	*
KI67	2.4 (0.85–6.5)	0.1	*	*
HER2	0.5 (0.26–0.98)	0.044	0.4322 (0.2186–0.8548)	0.01592
Tils	0.28 (0.17–0.46)	3.20E-07	0.2128 (0.1152–0.3931)	7.72e-07
MP class	0.65 (0.46–0.91)	0.013	*	*
Mitosis	1.4 (0.82–2.3)	0.23	*	*
Molecular	1 (0.69–1.5)	0.86	*	*

*Not statistically significant.

**Figure 2:**
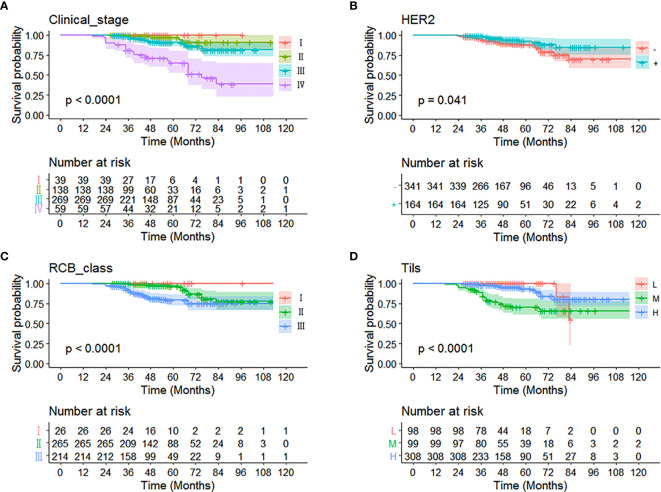
Kaplan-Meier curve of different independent prognostic factors. Multivariate analysis showed that **(A)** clinical stage, **(B)** HER2, **(C)** RCB grading, and **(D)** Tils were correlated with OS in non-PCR patients after neoadjuvant therapy for breast cancer.

The data were analyzed by substituting in a multivariate analysis, and the results were that clinical stage, HER2, RCB grading, and Tils grading were correlated with OS in non-PCR patients after neoadjuvant therapy for breast cancer. The 3-year survival of clinical stages I–IV were 100, 99.0, 98.5, and 81.3%; the 5-year survival of clinical stages I–IV were 100, 98.5, 93.3, and 69.5%; the HER2 negative or positive 3-year survival was 95.4, 99.1% and the 5-year survival was 90.7, 94.5%; the 3-year survival of RCB grades I, II, and III were 100, 99.4, and 92.6%; the 5-year survival of RCB grades were 100, 97.8, and 85.1%; and the 3-year survival and 5-year survival of Tils grades low, intermediate, and high were 77, 97, 99% and 69, 95, 99% (**Figure 2** and **Table 2**).

**Figure 3:**
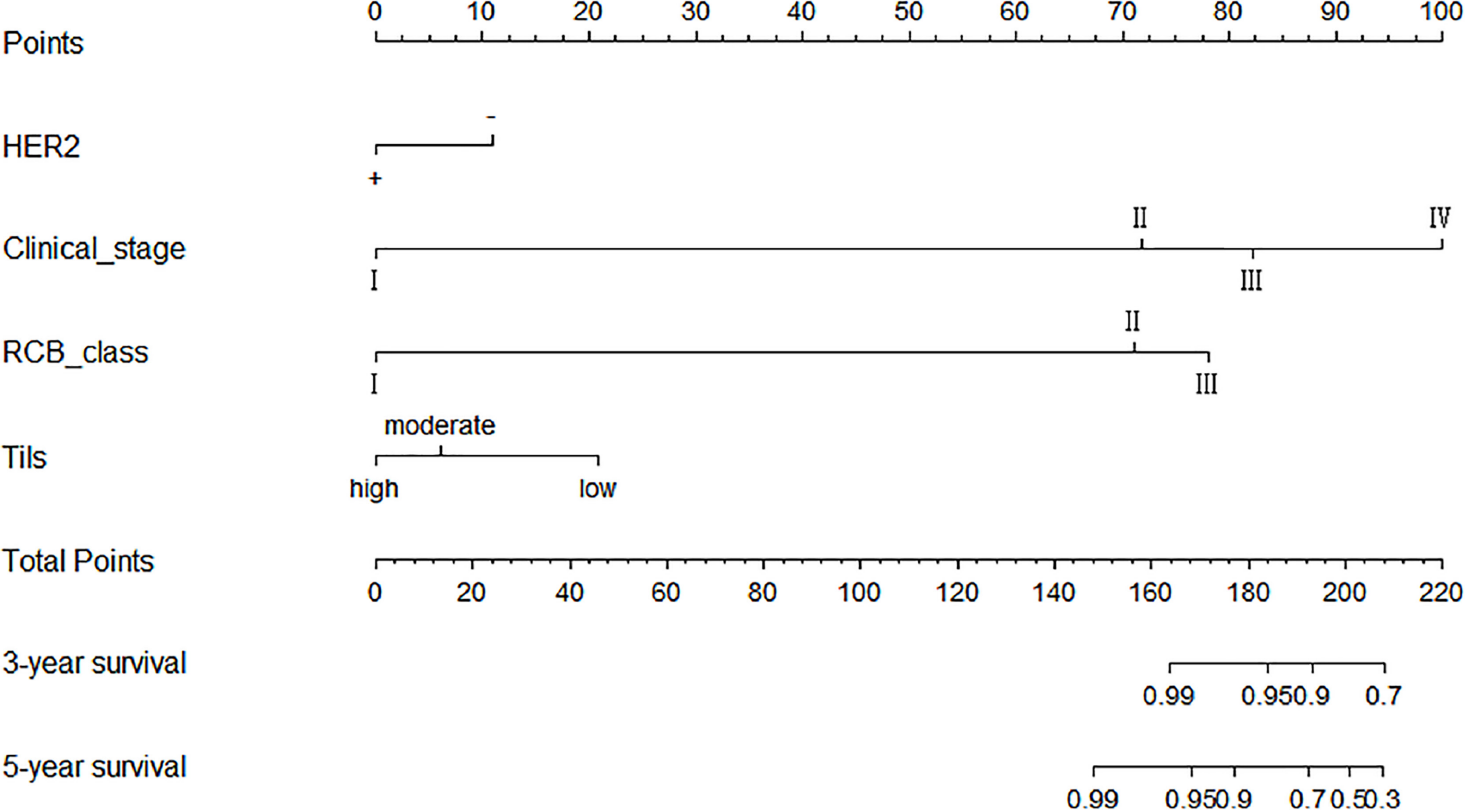
Schematic diagram of prediction model. Each factor corresponded to one point, the scores of the four factors were summed to give a total score, and finally the corresponding 3-year and 5-year survival rates were calculated by using the R software.

Significant indicators from the multivariate COX regression analysis were included in the prediction model development options, programmed using R software, and finally made into a prediction model based on clinical stage, HER2, RCB grading, and Tils grading system to assess the prognostic outcome of non-PCR patients after neoadjuvant therapy for breast cancer. Each factor corresponded to one Point, the scores of the four factors were summed to give a total score, and finally the corresponding 3-year and 5-year survival rates were calculated by using the R language formula (**Figure 3**).

**Figure 4:**
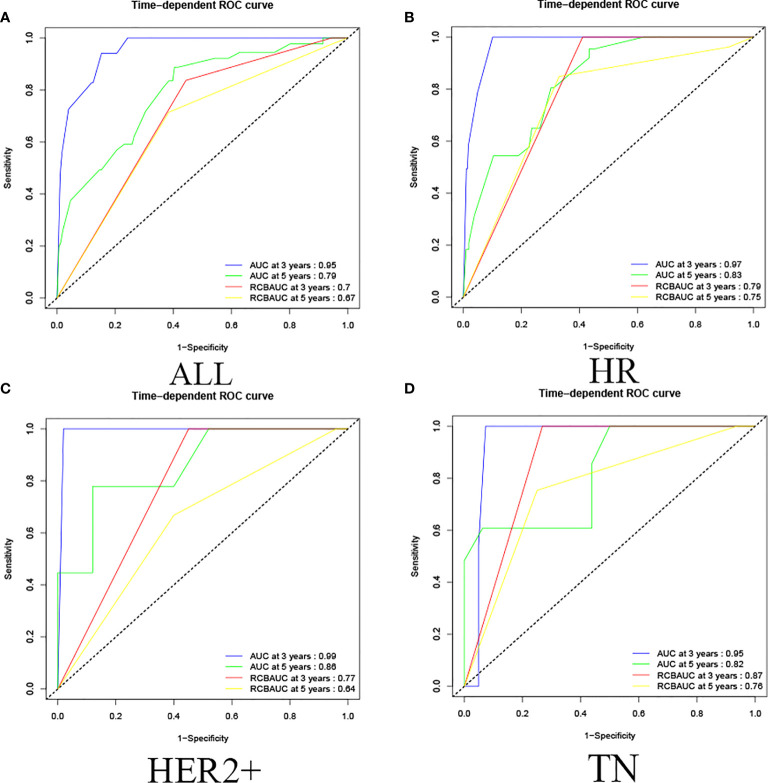
Training set ROC curve. **(A)** Among all cases in the training set, the prediction model predicted that the 3-year survival AUC value was 0.95 and the 5-year survival AUC value was 0.79, and the RCB classification of 3-year survival and 5-year survival were 0.70 and 0.67. **(B)** In HR, the AUC values of 3-year and 5-year survival rate calculated by predictive model were 0.97 and 0.83, the RCB classification of 3-year survival and 5-year survival were 0.79 and 0.75; **(C)** in HER2+, the AUC values of 3-year and 5-year survival rate calculated by predictive model were 0.99 and 0.86, the RCB classification were 0.77 and 0.64; **(D)** in TN, the AUC values of 3-year and 5-year survival rate calculated by predictive model were 0.95 and 0.82. The RCB classification were 0.87 and 0.76.

In this study, by using R language to calculate the prediction model calibration and ROC curve, among all cases in the training set, the prediction model predicted that the 3-year survival AUC value was 0.95 and 5-year survival AUC value was 0.79, and the RCB classification of 3-year survival and 5-year survival were 0.70 and 0.67, respectively, which proved that the prediction model could predict the OS of non-PCR patients after neoadjuvant therapy for breast cancer more accurately than the RCB classification, and showed the same results in HR, HER2+,and TN classifications. (In HR, the AUC values of 3-year and 5-year survival rate calculated by predictive model were 0.97 and 0.83; in HER2+ were 0.99 and 0.86; in TN were 0.95 and 0.82. In HR, the RCB classifications of 3-year survival and 5-year survival were 0.79 and 0.75; in HER2+ were 0.77 and 0.64; in TN were 0.87 and 0.76.) This study shows that the predictive model has a more accurate result than RCB classification in predicting OS of non-PCR patients after neoadjuvant chemotherapy for breast cancer in general and in each group (**Figure 4**).

**Figure 5:**
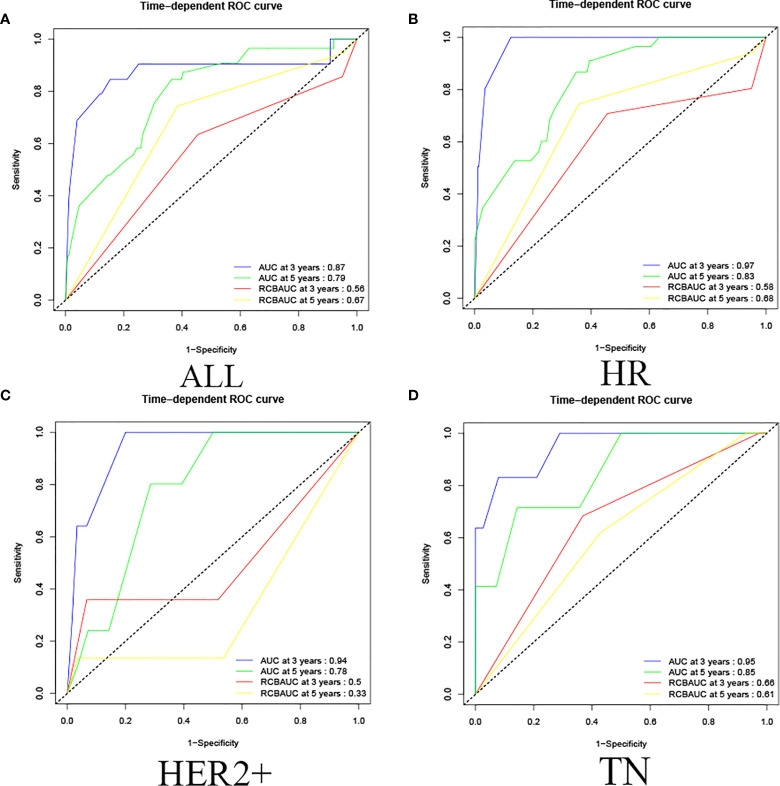
Validation set ROC curve. **(A)** Among all cases in the validation set, the prediction model of 3-year survival AUC value was 0.87 and the 5-year survival AUC value was 0.79, and the RCB classification of 3-year survival and 5-year survival were 0.56 and 0.67. **(B)** In HR, the AUC values of 3-year and 5-year survival rate calculated by the predictive model were 0.97 and 0.83, the RCB classification of 3-year survival and 5-year survival were 0.58 and 0.68; **(C)** in HER2+, the AUC values of 3-year and 5-year survival rate calculated by predictive model were 0.94 and 0.78, the RCB classification were 0.50 and 0.33; **(D)** in TN, the AUC values of 3-year and 5-year survival rate calculated by the predictive model were 0.95 and 0.85, and the RCB classification were 0.66 and 0.61.

The validated prediction model had a 3-year survival AUC value of 0.87 and a 5-year survival AUC value of 0.79 in all cases in the validation set, and showed the same results in HR, HER2+, and TN classifications. (In HR, the AUC values of 3-year and 5-year survival rate calculated by validated predictive model were 0.97 and 0.83; in HER2+ were 0.94 and 0.78; in TN were 0.95 and 0.85. In HR, the RCB classifications of 3-year survival and 5-year survival were 0.58 and 0.68; in HER2+ were 0.50 and 0.33; in TN were 0.66 and 0.61.) This study showed that the validated prediction model had accurate prediction results over RCB classification in predicting OS in non-PCR patients after neoadjuvant therapy for breast cancer in all cases in the validation set and in each classification (**Figure 5**).

**Figure 6:**
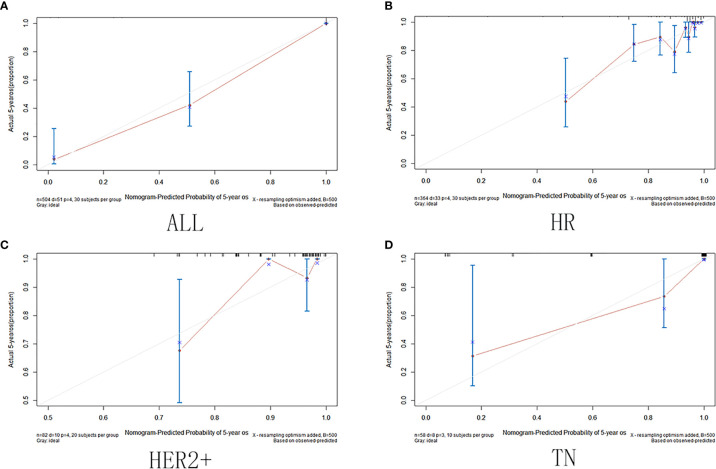
Training set calibration curve. **(A)** Among all cases in the training set; **(B)** in HR group; **(C)** in HER2+ group; **(D)** in TN group.

**Figure 7:**
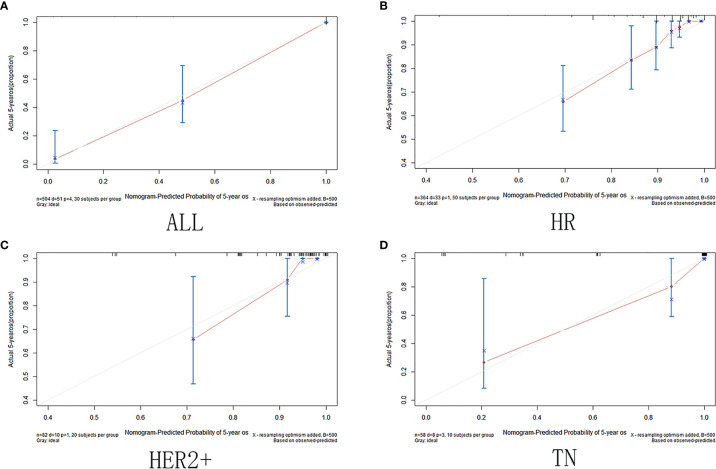
Validation set calibration curve. (**A**)among all cases in the validation set; **(B)** in HR group; **(C)** in HER2+ group; **(D)** in TN group.

The calibration curve shows that in the training and validation datasets, the prediction model can predict the OS of non-PCR patients after neoadjuvant treatment of breast cancer (**Figures 6** and **7**).

In this study, the 5-year survival rates were calculated from the training set and validation set data according to the R language formula, respectively, and divided into the >97, 90–97, 80–90, 70–80, 60–70, 50–60, and <50% groups, after which the number of people in each group was calculated against the actual number of deaths, and the actual survival rates were finally derived and compared to the predicted values. Through calculation, the results show that in all groups in the training set, the actual survival rate was consistent with the predicted 5-year survival rate interval, and the predicted 5-year survival rate is 98.6, 95.6, 86.5, 75.6, 66.7, 60.0, 21.4%, and the actual 5-year survival rate in the validation set was 98.7, 97.2, 88.8, 83.7, 61.5, 50.0, 35.7%. The results of the calculations showed that in all subgroups of the training and validation sets, the actual survival rates matched the predicted 5-year survival rate intervals, except for the 70–80% group, where the actual survival rates were higher than the predicted survival rates, and the actual 5-year survival rates in all other groups matched the predicted intervals. These data indicate that the predicted values of the prediction model developed in this study match the actual survival rates without underestimating the mortality risk and have a relatively accurate prediction effect (**Table 3**).

**Table 3:** Comparison of 5-year predicted survival rate and actual survival rate.

Training				Validation			
Predicted survival rate	Total	Death	Actual survival rate	Predicted survival rate	Total	Death	Actual survival rate
>97%	143	2	98.6%	>97%	176	2	98.7%
90–97%	183	8	95.6%	90–97%	141	4	97.2%
80–90%	104	14	86.5%	80–90%	107	12	88.8%
70–80%	41	10	75.6%	70–80%	49	8	83.7%
60–70%	15	5	66.7%	60–70%	13	5	61.5%
50–60%	5	2	60.0%	50–60%	4	2	50.0%
<50%	14	11	21.4%	<50%	14	9	35.7%

The results of the calculations showed that in all subgroups of the training and validation sets, the actual survival rates matched the predicted 5-year survival rate intervals, except for the 70–80% group, where the actual survival rates were higher than the predicted survival rates.

To predict the prognostic outcome of non-pCR patients after neoadjuvant therapy for breast cancer and to provide treatment protocols for patients undergoing surgery after neoadjuvant therapy, this study compared data with 5-year survival rate predicted by the prediction model >97% actual survival rate. In the training set, the prediction model predicted that the number of people with 5-year survival rate >97% was 143, the actual number of deaths was 2, and the actual survival rate was 98.6%. In the HR, HER2+, and TN groups, the actual survival rate of patients with predicted survival rate >97% was 99, 97, and 100%, respectively. In the validation set, the prediction model predicted that the number of 5-year survival >97% was 176, the actual number of deaths was 2, and the actual survival rate was 99%. In the HR, HER2+, and TN groups, the actual survival rate of patients with a predicted survival rate of >97% was 99, 98, and 100%, respectively. Comparison of the predicted results with the actual results showed that stratifying the predicted survival and predicting survival >97% both showed predictions that matched the actual results. This indicates that the prediction results of the prediction model match the actual results with high agreement, and can be used to predict the prognostic outcome of non-PCR patients after neoadjuvant therapy for breast cancer and provide an assistance to subsequent clinical treatment (**Table 4**).

**Table 4:** Compared data with 5-year survival rate predicted by the prediction model >97% actual survival rate.

Training				Validation			
Group	Total	Death	Actual survival rate	Group	Total	Death	Actual survival rate
HR	99	1	99%	HR	118	1	99%
HER2+	33	1	97%	HER2+	45	1	98%
TN	11	0	100%	TN	13	0	100%

HR, hormone receptor positive; HER2+, human epidermal growth factor 2 positive; TN, triple-negative breast cancer. This indicates that the prediction results of the prediction model match the actual results with high agreement.

## Discussion

In recent years, the incidence of female breast cancer has increased rapidly worldwide and breast cancer has become the most prevalent malignancy in women (17). It affects women of all ages and races, so research into the treatment and prognostic factors of breast cancer is crucial. Firstly, neoadjuvant therapy for certain patients whose masses are too large or who are otherwise inoperable, so as to downstage their cancer to meet the criteria for surgery or to control disease progression and thus prolong survival, is now widely applied in the treatment of breast cancer due to its remarkable results (18–20). pCR is a recognized endpoint in clinical trials of neoadjuvant therapy, and many studies have shown that pCR is independently correlated with improved survival outcomes compared to non-pCR patients, and thus can serve as a useful prognostic indicator (21). In order to investigate the factors correlated with prognosis in non-pCR patients, predict the prognosis of patients after neoadjuvant therapy, and provide a basis for clinical decisions on further treatment, cases that did not achieve pCR after neoadjuvant therapy for invasive breast cancer were selected for study and prognostic modeling in this study to provide prognostic prediction results as well as a basis for further treatment.

In this study, RCB, Tils, HER2, and clinical stage were selected as prognostic factors correlated with overall survival by univariate and multivariate survival analyses. Univariate analysis showed that clinical stage was negatively correlated with prognostic outcome, and the higher the clinical stage, the worse the prognosis and the shorter the overall survival, which is consistent with other studies. Tils have been extensively validated as an independent prognostic factor in breast cancer patients in recent years, especially for patients with HER2-positive breast cancer and triple-negative breast cancer. Studies have shown that Tils, as a continuous variable, are significantly correlated with prognosis. By setting a cutoff value for Tils and analyzing the prognostic outcomes of patients above and below the cutoff value, the results showed that higher expression of Tils was correlated with better prognostic outcomes. This is consistent with the results of our study. (22, 23) However, some studies have shown that the prognostic prediction effect of Tils was not satisfactory for HER2-negative patients (24). Therefore, more prognostic factors need to be combined in order to achieve better prediction effect.

HER2 expression is present in approximately 1/4 of breast cancer cases, and HER2-positive expression has been shown to be correlated with a worse prognosis. In order to prolong the survival of HER2-positive breast cancer patients, HER2 has been shown to be an effective therapeutic target for the treatment of breast cancer (25). The present study showed that patients with HER2-positive expression before neoadjuvant therapy had a better prognosis, which was correlated with the better outcomes brought by the HER2-targeted therapy, and this is consistent with the results of some studies in HER2-positive breast cancer (26).

The interpretation of the residual tumor burden scoring system after neoadjuvant therapy uses four indicators: tumor bed size, proportion of invasive cancer, number of lymph node metastases, and maximum diameter of lymph node metastases after neoadjuvant therapy. The inclusion of more factors and a more comprehensive residual tumor burden scoring system were better validated, with an increased residual tumor burden score correlated with a worse prognosis and shorter survival time. This result was validated in this and other studies (7, 27).

Because pathological factors alone have limitations in predicting the outcomes after neoadjuvant therapy for breast cancer, and because the impact of pathological factors on prognosis is changing as technology continues to advance and treatments continue to improve, there is a need for better models to predict patient prognosis for better prognostic outcomes with further treatment. Some studies have shown that the combination of RCB and KI67 or the combination of RCB and Tils could obtain better predictions than the RCB system (8, 28). These studies illustrate that selection of broader and more meaningful clinicopathological information can lead to the development of more accurate prediction models. Other studies had different limitations. Most only combined two indicators, and someone screened cases with triple-negative or HER2-positive molecular subtypes after neoadjuvant therapy. Therefore, these researches showed inevitable limitations on the application.

We produced a new prediction model to assess the prognosis of non-PCR patients after neoadjuvant therapy by analyzing four prognostic factors selected and combining the four prognostic factors by R software. We validated the model by calibration plots, ROC curves, and comparison of predicted results with actual results. The prediction effect of the model was validated by ROC curves, and the prediction model AUC values were 0.75–0.97 for all data in the training and validation sets and for data of each subgroup, showing a more accurate predictive ability than RCB alone. For the calibration plots, the calibration curves fit the ideal state, indicating that the prediction model can well predict OS in non-PCR patients after neoadjuvant therapy for breast cancer in both the training and validation datasets. Our prediction model analyzed all molecular types of cases after neoadjuvant therapy, selected more comprehensive influencing factors, and had a good verification effect on all molecular subtypes after neoadjuvant therapy, which made the model more effective and extensive on the application. We found that the predicted survival rate was coincident with actual survival rate among groups after comparing, except for the validation set 70–80% group, which the actual survival rate was slightly higher than the predicted survival rate. Therefore, we think, this study developed a new predictive model for the prognosis of non-pCR patients after neoadjuvant treatment of breast cancer, which applied to all patients and will not underestimate the survival risk of patients.

On the basis of the predicted results of the model, clinicians can individualize the patient’s further treatment. For patients with lower risk, routine treatment or intensity-reduction treatment could be used to reduce the side effects. For patients with high risk, high-intensive treatment can be appropriately increased in further process to prolong the survival time and reduce the survival risk as much as possible.

Targeted therapy has been the main research direction in tumor therapy; meanwhile, researches on molecular markers has become hotspot. But in this study, because of the limitations of samples, we only included HER2 into the analysis. Subsequently, we will bring molecular biomarkers such as PD-L1, BRCA, and NGS into our research to optimize model performance.

In conclusion, we developed a more accurate predictive model than RCB by combining four breast cancer prognostic factors using R software to assess the prognosis of non-pCR patients after neoadjuvant therapy for breast cancer and to provide a basis for clinical decision-making for further treatment.

## Data Availability Statement

The original contributions presented in the study are included in the article/supplementary files. Further inquiries can be directed to the corresponding author at (15954713520@163.com).

## Ethics Statement

The studies involving human participants were reviewed and approved by the Ethics Committee of the Fourth Hospital of Hebei Medical University. Written informed consent for participation was not required for this study in accordance with the national legislation and the institutional requirements.

## Author Contributions

YY completed the article design, data statistics, and article editing throughout the entire process. All authors participated in the writing of the article. All authors contributed to the article and approved the submitted version.

## Conflict of Interest

The authors declare that the research was conducted in the absence of any commercial or financial relationships that could be construed as a potential conflict of interest.

## Publisher’s Note

All claims expressed in this article are solely those of the authors and do not necessarily represent those of their affiliated organizations, or those of the publisher, the editors and the reviewers. Any product that may be evaluated in this article, or claim that may be made by its manufacturer, is not guaranteed or endorsed by the publisher.
